# Implantable Intracranial Pressure Sensor with Continuous Bluetooth Transmission via Mobile Application

**DOI:** 10.3390/jpm13091318

**Published:** 2023-08-28

**Authors:** Yasmeen Elsawaf, Erik Jaklitsch, Madison Belyea, Levon Rodriguez, Alexandra Silverman, Halyn Valley, Issam Koleilat, Nasser K. Yaghi, Michael Jaeggli

**Affiliations:** 1 Department of Neurological Surgery, Oregon Health & Science University, Portland, OR 97201, USA; 2 Department of Biomedical Engineering, Northeastern University, Boston, MA 02115, USA; 3 Department of Surgery, Community Medical Center, RWJ/Barnabas Health, Toms River, NJ 08753, USA; 4 Department of Neurosurgery, Barrow Neurological Institute, Phoenix, AZ 85013, USA; nasser.yaghi@barrowbrainandspine.com

**Keywords:** intracranial pressure monitor, ventriculoperitoneal shunt, hydrocephalus, shunt failure, neurosurgical innovation

## Abstract

Hydrocephalus is a clinical disorder caused by excessive cerebrospinal fluid (CSF) buildup in the ventricles of the brain, often requiring permanent CSF diversion via an implanted shunt system. Such shunts are prone to failure over time; an ambulatory intracranial pressure (ICP) monitoring device may assist in the detection of shunt failure without an invasive diagnostic workup. Additionally, high resolution, noninvasive intracranial pressure monitoring will help in the study of diseases such as normal pressure hydrocephalus (NPH) and idiopathic intracranial hypertension (IIH). We propose an implantable, continuous, rechargeable ICP monitoring device that communicates via Bluetooth with mobile applications. The design requirements were met at the lower ICP ranges; the obtained error fell within the idealized ±2 mmHg margin when obtaining pressure values at or below 20 mmHg. The error was slightly above the specified range at higher ICPs (±10% from 20–100 mmHg). The system successfully simulates occlusions and disconnections of the proximal and distal catheters, valve failure, and simulation of A and B ICP waves. The mobile application accurately detects the ICP fluctuations that occur in these physiologic states. The presented macro-scale prototype is an ex-vivo model of an implantable, rechargeable ICP monitoring system that has the potential to measure clinically relevant ICPs and wirelessly provide accessible and continuous data to aid in the workup of shunt failure.

## 1. Introduction

Hydrocephalus is a clinical disorder characterized by excessive accumulation of cerebrospinal fluid (CSF) within the ventricles of the brain due to an obstructive process or disturbance to the normal absorption pathways within the ventricular system. Several CSF diversion techniques exist to redirect the excess fluid from the ventricles to areas of the body with greater lymphatic absorption. The most common diversion technique is a ventriculoperitoneal (VP) shunt; this device allows CSF to drain from the ventricles to the peritoneum. Other common devices include ventriculopleural, ventriculoatrial, or lumboperitoneal shunts. 

Forty percent of shunts fail within two years after implantation due to obstruction, disconnection, or equipment failure, such as valve or catheter malfunction [1]. Shunt failure in most shunt-dependent patients (except in NPH, low compliance ventricular systems, and other rare etiologies) causes elevated intracranial pressures. Shunt failure workup currently employs mainly invasive measures, such as shunt tapping or exploratory revisions. Additionally, current neuromonitoring technology does not allow for remote ICP measurement; the gold standard involves invasive insertion of an intraparenchymal pressure monitor or external ventricular drain to obtain ICP measurements and/or drain CSF if indicated. Physiologic ICP varies per patient, though generally tolerable ICP ranges from 0–20 mmHg; acute sustained elevations in ICP to 20–40 mmHg may yield symptoms and warrant intervention [2,3,4,5]. When monitoring ICP, waveforms (A, B, C) may indicate underlying pathology but may also correlate with normal changes in arterial blood pressure, respiratory rate, and sleep cycle [4,6,7]. In A waves, the ICP rises steeply from normal pressure (approximately 10 mmHg) to 50 mmHg or higher. The pressure remains elevated for 5–20 min and then drops sharply. In B waves, the ICP rhythmically oscillates every 1–2 min between normal pressure and 30–40 mmHg. In C waves, the ICP rhythmically oscillates with a relatively small amplitude 4–8 times per minute [6]. Though patient dependent and not definitive, A and B waves may be indicative of abnormal pathology, prompting a diagnosis of shunt failure. 

Currently, shunt failure diagnosis often requires an invasive inpatient workup as there is no fully internally implanted device that directly measures ICP, providing accessible and continuous ICP readings to assist in a noninvasive diagnosis of shunt failure [3,4,8,9,10]. To address the unmet need, we present our macro-scale ex-vivo prototype for an implantable, continuous, rechargeable ICP monitoring system that communicates wirelessly (via Bluetooth) with patients’ iOS devices. This device aims to enhance patient care and autonomy by providing continuous feedback of shunt function. Additionally, this device will help aid the study of disease pathologies such as normal pressure hydrocephalus (NPH) and idiopathic intracranial hypertension (IIH). 

## 2. Materials and Methods

The prototype was built within the Biomedical Engineering labs at Northeastern University in Boston, MA, USA. The supplies utilized include a Medtronic Strata valve (Minneapolis, MN, USA), Codman proximal and distal catheters (Princeton, NJ, USA), a 2FMV microelectromechanical (MEMS) Connect 100 Vented Millar, Inc. Piezoresistive Sensor with a 0.67 mm outer diameter (Houston, TX, USA), Arduino Nano 33 BLE microcontroller (Somerville, MA, USA), 2 GB microSD and adapter, 2500 mAh lithium-ion battery, water column, runoff ramp. These supplies were selected considering key design requirements and employing a representative trade study.

### 2.1. Key Design Requirements

The four key design requirements consisted of specific measurements for pressure sensing, data collection, safety, and wireless transmission.

1. Pressure Sensing: The system must measure ICP within a range of 0–100 mmHg (±2 mmHg from 0–20 mmHg and ±10% from 20–100 mmHg);
2. Components: The system must wirelessly transmit data to a mobile device at a frequency of 2 Hz without compromising safety and store 54 mB of data locally;
3. Safety: The system must be powered by a rechargeable battery that meets industry safety requirements, and information security must be addressed to ensure patient data safety;
4. Mobile Application: The application must display continuous ICP readings and detect A and B ICP waveforms and acute elevations in ICP.

### 2.2. Trade Study and Design Solutions

A representative trade study was initially conducted to evaluate the most optimal design components for pressure sensing methods and location, methods of wireless transmission, powering options, storage capabilities, and integration of the system components, as well as logistical elements such as durability and attainability. Table 1 details the trade study results; see Appendix A for the trade study report.

## 3. Results

### 3.1. Prototype Development and Testing

#### 3.1.1. Prototype Development

**Figure 1:**
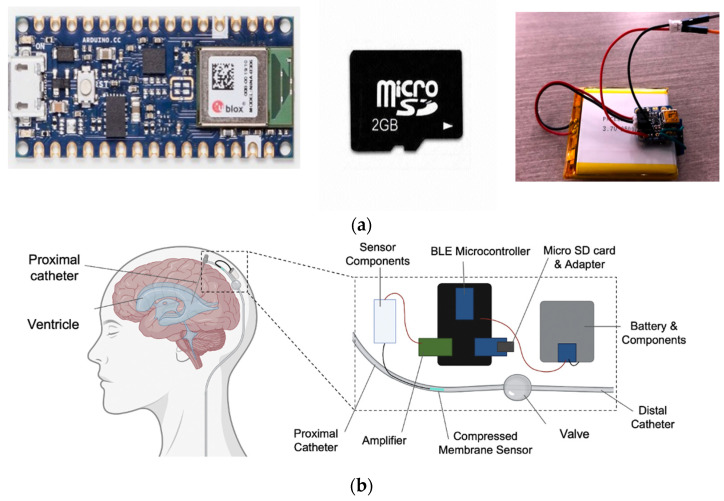
(**a**) Prototype components (left to right: microcontroller, 2 GB microSD, and 2500 mAh Lithium Ion Battery). (**b**) Macro-scale prototype demonstrating the ventriculoperitoneal shunt system (proximal catheter in the ventricle, a sensor within the lining of the proximal catheter, in line with BLE microcontroller on the surface of the skull with attached sensor components, micro SD/adapter, battery and components, and amplifier all proximal to the valve and distal catheter.

A provisional patent was obtained prior to the development of the prototype (U.S. Prov. Patent, 63/093,154). A macro-scale prototype of the device was created utilizing the 2FMV microelectromechanical connect 100 vented Millar piezoresistive pressure sensor, an Arduino Nano 33 BLE microcontroller to integrate the system and transmit pressure valves to the iOS application, a 2 GB microSD card to provide backup storage, and an implantable, 2500 mAh Lithium-Ion battery recharged with Qi technology to power the system as demonstrated in Figure 1a,b. Qi is an established method of wireless power transmission which utilizes inductive coupling; the external Qi transmitter coils in the wireless charger aligns with the implanted Qi receiver coils in the device to establish a connection and transfer power [11]. This charging modality has since been widely implemented in many health monitoring devices, such as wearable heart rate monitors [12]. 

#### 3.1.2. Prototype Testing

**Figure 2:**
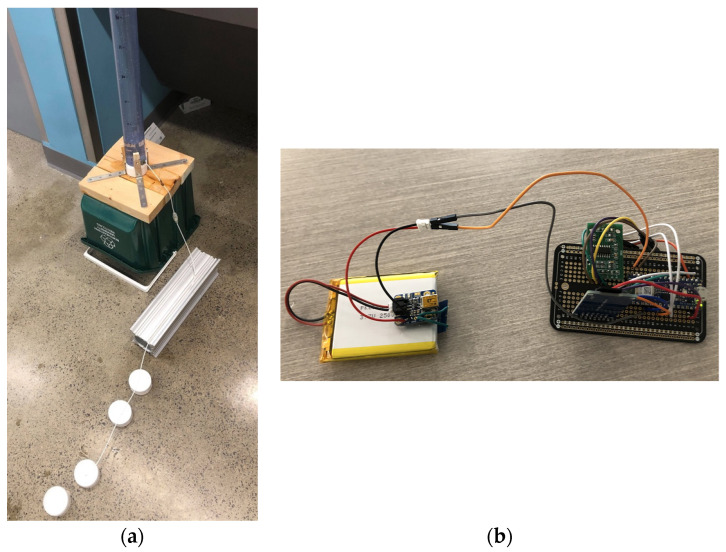
(**a**) Prototype testing setup: water column with the proximal catheter inside and Millar sensor within the proximal catheter, in line with the Strata valve, distal catheter on run-off ramp with collection system (**b**) circuit board setup: Arduino Nano 33 BLE microcontroller, 2 GB microSD and Adapter, 2500 mAh Lithium-Ion battery.

Prototype testing was performed utilizing a water column to generate physiologically relevant pressures and mimic the environment of the CSF filled ventricles. The proximal catheter was inserted into the bottom of the water column. To mimic the idealized miniaturized pressure sensor integrated into the lining of the catheter, a needle was used to create a pinpoint hole in the proximal catheter, and the pressure sensor was inserted inside the catheter, pointing toward the direction of flow. The distal end of the proximal catheter was connected to the proximal end of the valve in series, and the proximal end of the distal catheter was connected to the distal end of the valve in series. The distal end of the catheter was placed in a runoff ramp with a collection system, as depicted in Figure 2a. The distal end of the pressure sensor was connected in series to the amplifier on the circuit board. Within the circuit board, the Arduino Nano 33 BLE microcontroller, 2 GB microSD and adapter, and 2500 mAh lithium-ion battery (placed under the circuit board) were connected separately Figure 2b. 

#### 3.1.3. Prototype Calibration

**Figure 3:**
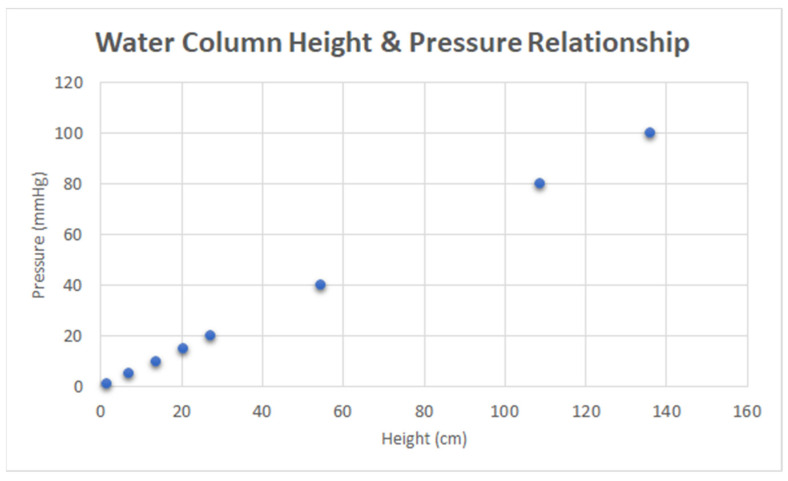
Calibration data: water column height and pressure relationship.

Bernoulli’s equation $\left(P+\frac{1}{2}{\rho V}^{2}+\rho gh=constant\right)$ was used to determine the height of water associated with each pressure value. Of note, the velocity term was considered negligible because the volume of water flowing out of the system was simultaneously replenished within the column. As a result, the measured gauge pressure was linearly proportional to the height of water (∆P=ρgh), as depicted in Figure 3, where the x-axis indicates the height of water in cm, and the y-axis indicates pressure in mmHg. 

**Figure 4:**
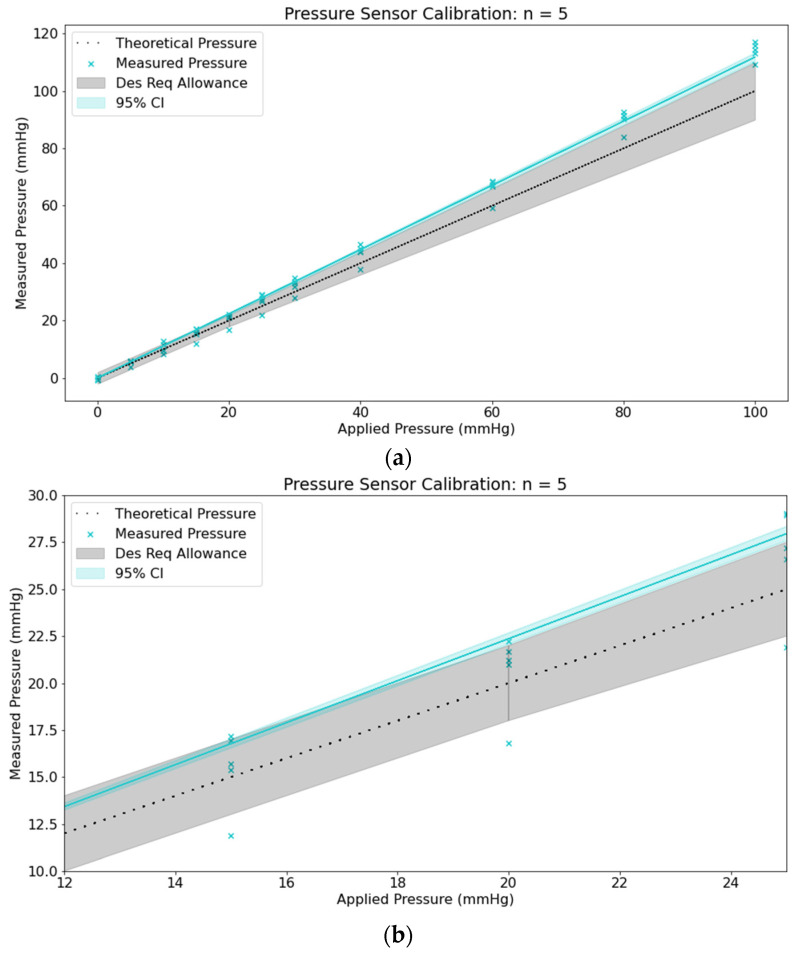

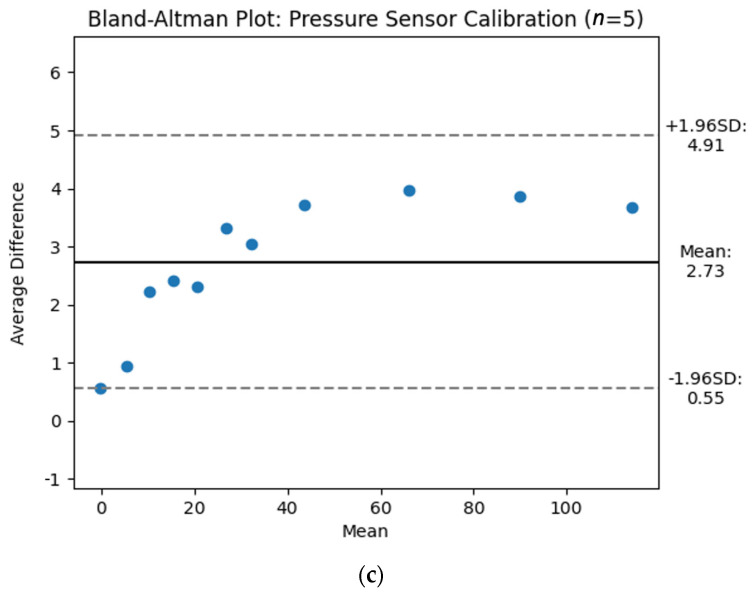
(**a**) Micro and (**b**) macroscale of pressure sensor calibration graph depicting the error allowance of ±2 mmHg from 0–25 mmHg, and ±10% from 25–100 mmHg (**c**) Bland–Altman plot displaying mean difference (*n* = 5) of measurements versus mean.

The pressure was calibrated from 0 to 100 mmHg by acquiring 60 data points at each theoretical pressure and repeating this process five times. The black dotted line in Figure 4a,b is the theoretical result, the grey area represents the error allowance of ±2 mmHg from 0–20 mmHg and ±10% from 20–100 mmHg. The teal line is the fitted linear regression through the origin, and the teal-shaded portion is the 95% confidence interval. Additionally, a Bland–Altman analysis was conducted for pressure sensor calibration (Figure 4c). Bland–Altman plots, conventionally, serve to compare measurements across two methods, plotting the difference between an average of these two measurements. In this instance, there are five measurements (*n* = 5) to be compared. These axes display the mean difference between all five measurements (y-axis) and the mean of all five measurements (x-axis). “Mean difference between all five measurements” refers to calculating all differences between the datasets (for *n* = 5 datasets, this is ten differences total) and averaging those values.

#### 3.1.4. Prototype Modeled ICP Detection

Pressure data was obtained utilizing the water column system as previously described. Successful pressure data was obtained between 0 and 100 mmHg with wireless transmission at a frequency of 2 Hz and confirmed accuracy at lower pressure ranges. Five calibration trials were conducted to validate the accuracy of the water column system. The obtained error of the device fell within the idealized ±2 mmHg margin when obtaining pressure values at or below 20 mmHg. At 25 mmHg, the obtained pressures slowly deviated from the design requirement allowance, with progression through higher pressure values. Table 2 provides a data set for these trials; the attached supplement provides average attained pressure values for each theoretical pressure.

**Figure 5:**
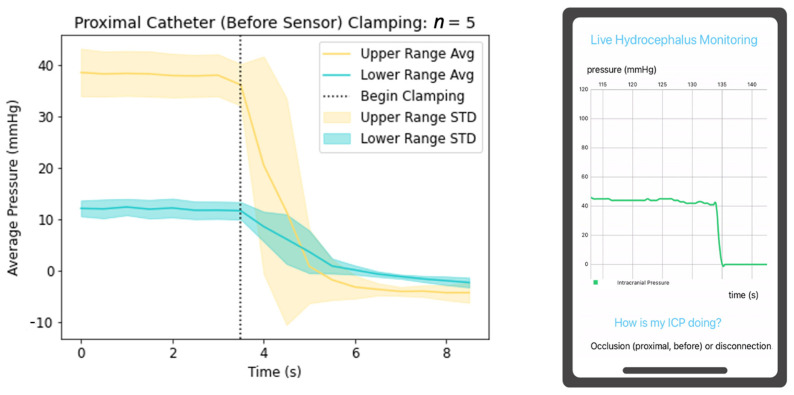
Depiction of ICP changes during proximal catheter clamping and mobile application detection of the physiologic state.

**Figure 6:**
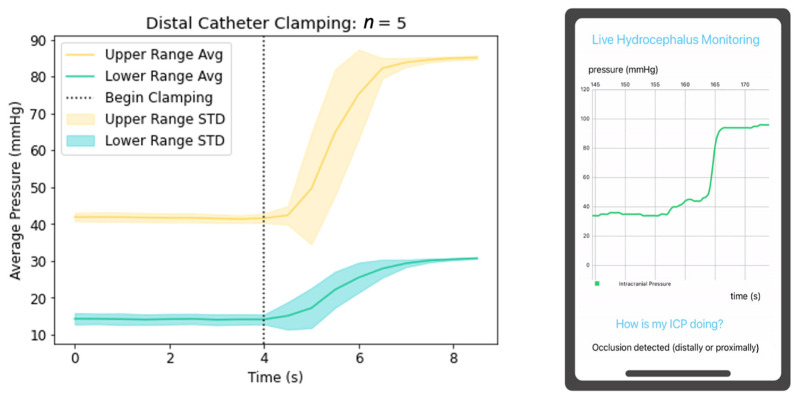
Depiction of ICP changes during distal catheter clamping and mobile application detection of the physiologic state.

The prototyped system also successfully detects occlusions and disconnections of the proximal and distal catheter, as well as physiologically abnormal A and B ICP waves. Occlusion and disconnections of the system were simulated by clamping the catheter at different locations in the validation setup and measuring the change in pressure; this process was repeated five times. Clamping the catheter proximal to the pressure sensor mimics physiologic conditions such as occlusion of the proximal catheter or valve setting changes, yielding decreased resistance and increased CSF drainage. In the proposed system, the pressure sensor lies within the catheter wall at the most distal end (near the valve connection) rather than in the proximal catheter within the ventricles; thus, proximal occlusion yields ICPs of zero rather than reflecting true elevated ICPs. The graphical depiction of the process is demonstrated in Figure 5, with high initial pressure graphed in yellow and low initial pressure graphed in blue. The average pressure is shown as a solid line with the standard deviation in the shaded region. The vertical dotted line indicates when clamping began. The mobile application then successfully detects this change and notifies the user of this change. Further modifications of the mobile application will include programming to prompt users when significant declines in pressure occur, as this situation may reflect truly elevated ICPs depending on pressure sensor placement within the catheter. Clamping the proximal catheter distal to the pressure sensor yields true ICP elevations, paralleling physiologic conditions such as valve failure or valve setting changes yielding increased resistance and less CSF drainage, or distal catheter occlusion yielding impairment of CSF outflow into the peritoneum as demonstrated in Figure 6. 

**Figure 7:**
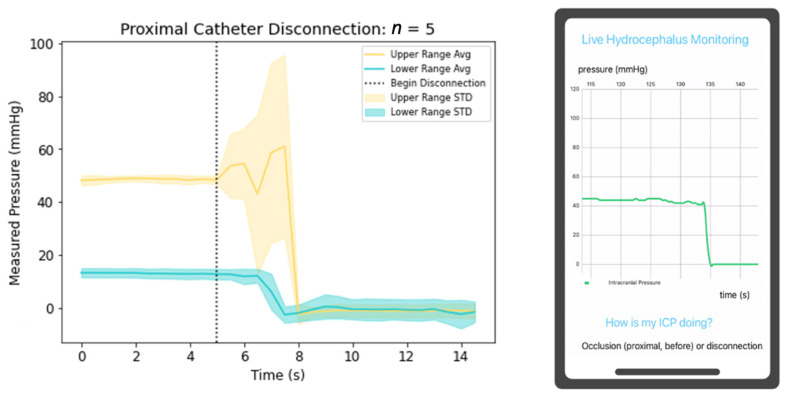
Depiction of ICP changes during proximal catheter disconnection and mobile application detection of the physiologic state.

**Figure 8:**
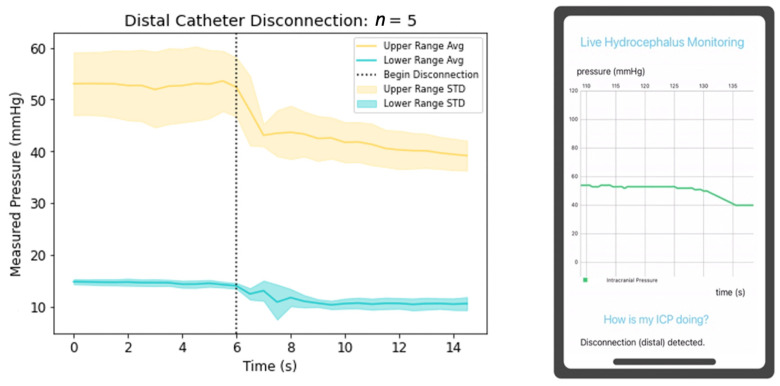
Depiction of ICP changes during distal catheter disconnection and mobile application detection of this physiologic state.

Catheter fracture or disconnection also results in VP shunt failure; to model this, the proximal and distal catheters were separately disconnected from the valve, and the resultant pressure changes were detected; this process was repeated five times at initial high and low ICPs. When disconnecting the proximal catheter from the valve, the resultant ICP value was zero Figure 7. This physiologically may initially yield lower ICPs due to a circuit with decreased resistance, though after the development of a pseudomeningocele (subcutaneous CSF collection) and increased resistance without an outlet for outflow, CSF will build up within the ventricles yielding elevated ICPs. Distal disconnection of the catheter from the valve yields a reduction of the ICP, though not completely to zero as shown in Figure 8. This clinical scenario yields variable effects depending on the presence of a calcified tract where CSF may continue to drain to the peritoneum, pleura, or atrium. If not present, the disconnection will yield elevated ICPs as resistance will rise at the disconnection point, and drainage will decrease. Though it is impossible for the in-vitro water-column setup to precisely mimic physiological conditions, our mobile application successfully recognized physiologic states simulated with the experimental data.

**Figure 9:**
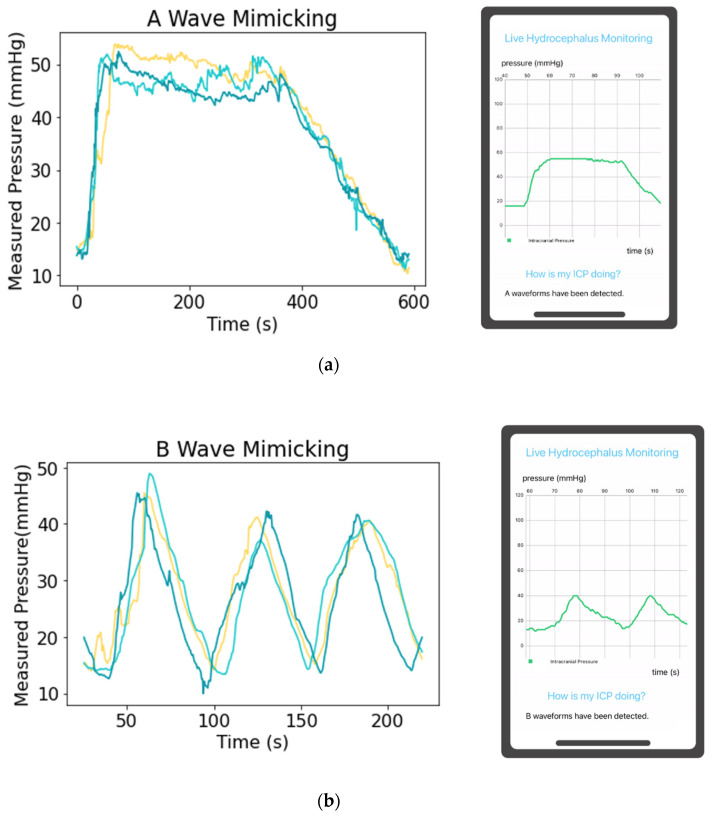
Depiction of (**a**) ICP A and (**b**) ICP B waves and mobile application detection of these physiologic states.

A utility of the proposed system is the ability to recognize dangerous ICP waveforms, though subject to patient physiology and underlying diagnosis. It is important to note that our system was unable to simulate physiologic P1, P2, and P3 waves and, thus, are not reflected in our ICP graphs. These waves correlate with arterial pulse. P1, the percussion wave, correlates with the arterial pulse transmitted through the choroid plexus into the CSF and via a column of fluid into the EVD transducer. P2, the tidal wave, represents cerebral compliance. P3, the dicrotic wave, correlates with the closure of the aortic valve. We were able to replicate “A” waves, as well as “B” and “C” waves, to demonstrate a steady state of ventricular compliance or lack thereof in A and B waves. A waves may be clinically relevant measures of ICP performance, during which a patient’s ICP rises from a baseline to around 50 mmHg, where it plateaus before returning to baseline values shown in Figure 9a. B waves are characterized by oscillations in ICP peaking at pressures greater than 40 mmHg, as displayed in the referenced Figure 9b. The proposed system successfully mimics A and B waves, and the mobile application recognizes both physio-logically relevant waves as depicted in the mobile application interface.

#### 3.1.5. Prototype Battery and Data Safety

**Figure 10:**
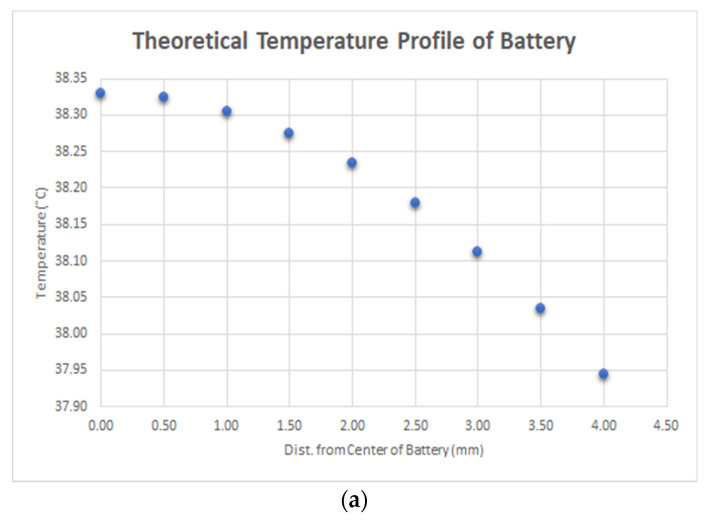

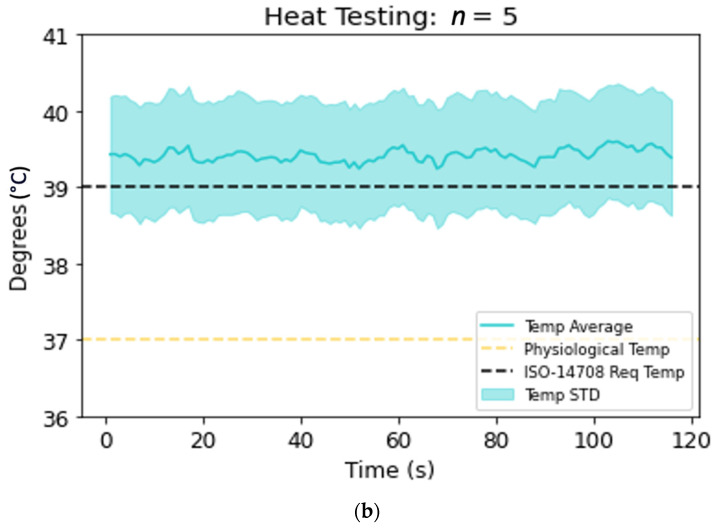
(**a**) Theoretical temperature profile of battery and (**b**) battery heat testing through thermal imaging.

Battery safety of the 2500 mAh lithium-ion battery was mathematically modeled and experimentally evaluated. According to ISO standard 14708, the outer surface of the implanted battery cannot exceed 39 °C [13]. A 1D-shell analysis at steady state was performed utilizing the equation $Tbatt\;\left(x\right)=-\frac{Q_{gen,batt}}{2k_{batt}}x^{2}+38.3\;{}^{\circ}C$ while charging under 3.7 V and a 0.5 A current; the surface of the battery was estimated to be just below 38 °C. One-dimensional steady state refers to temperature as a function of a single dimension or spatial coordinate, for which heat flow is constant with time [14]. The temperature profile demonstrates a negative linear relationship between the temperature of the battery and the distance from the center of the battery shown in Figure 10a; this distance is along the coordinate that measures battery thickness. Battery temperature was also evaluated through thermal imaging. The temperature of the battery was calculated while charging inside an incubator at 37 °C. The data depicted in Figure 10b demonstrates that the average temperature falls slightly above 39 °C; the industry standard falls within one standard deviation of these measurements. The design requirements for battery safety were not obtained. Considerations include the inability to mimic exact physiologic conditions within the incubator; namely, the specific heat of air differs from that of biological tissue. Additionally, continuous heat transfer in the incubator occurs through convection rather than conduction, as it would when implanted subcutaneously. 

In the initial models of the mobile application, data will be stored and saved locally on users’ mobile devices. The application was created utilizing X-code, which includes Data Vault, an Apple owned data storage mechanism with basic security features. Additionally, in future iterations of the device we aim to integrate data into electronic medical records equipped with HIPAA (Health Insurance Portability and Accountability Act) compliant encrypted databases to avoid data breaches. Furthermore, it is technically feasible to avoid integrating PHI (personal health information) into the application and create de-identified patient codes to avoid recognition of individual patient’s pressure readings. If patient data is integrated into the application, these indexed demographics will be hashed (mapping data as an integer value) to prevent data from being accessible to third parties. Additionally, it is important to note that in the current iteration of the device it is not possible for users to modify the settings of their valve via the mobile application; thus, external access of the device cannot yield physical harm to patients.

In making this data accessible to the patient and physician, the design requirements of differentiating between A waves, B waves, and normal fluctuations in ICP as well as notifying patients of dangerous ICPs were satisfied. The mobile application depicts real time ICP data and shunt functionality that users can navigate to within one click. Lastly, the application is iOS compatible and stores all data transmitted by Bluetooth from the system.

## 4. Discussion

The prototyped device presented here offers a technological advancement to augment the current standard of care for patients with implanted shunts and assist in noninvasive triage of shunt failure. This Bluetooth compatible wireless intracranial pressure monitor has the potential to improve the quality of care provided to patients with lifelong shunts, which are prone to a high failure rate, while also improving patients’ quality of life by providing autonomy over their device. Likewise, this system can provide significant insights into difficult to understand pathophysiologic states that center around CSF fluid dynamics, such as in the disease states of normal pressure hydrocephalus (NPH) and idiopathic intracranial hypertension (IIH). 

Existing intracranial pressure monitors include those utilizing invasive pressure and CSF flow dynamics as well as wearable and wireless CSF flow monitoring. Those utilizing microelectromechanical systems (MEMS) technology, like our presented device, include the OSAKA telesensor, the MIETHKE Sensor Reservoir, and the Raumedic Neurovent P-tel probe. The OSAKA telesensor measures absolute ICP using a resonant circuit containing a capacitor and coil. The intracranial pressure is transmitted in a vacuum through the bellows of the device and to the ferrite core. The ferrite core then changes the resonant frequency, causing pulse waves, which are measured by an external probe at a rate of 50 times per second. This telesensor measures a pressure range of −25 mmHg to +75 mmHg with an accuracy of ±1.5% and a sensitivity of 0.2 mmHg [15]. The MIETHKE Sensor Reservoir is a type of telemetric ICP monitoring device that consists of two parts: the implanted sensor reservoir and the external pressure reader. It is possible to integrate the sensor reservoir with a shunt system by placing the sensor into the burr hole, where the bottom outlet of the reservoir projects into the brain parenchyma. The sensor reservoir is 23.8 mm in diameter and 7.7 mm in height, and consists of a silicone dome encapsulated in a housing made of polyether ether ketone. To measure intracranial pressure, the sensor also contains a measuring cell which is covered in titanium and integrated with 64 different pressure sensors. When cerebrospinal fluid enters the device through the bottom outlet, the intracranial pressure is transmitted to the titanium measuring cell, which is detected and quantified by the pressure sensors. To obtain the intracranial pressure, the external pressure reader must be placed directly above the reservoir and the intracranial pressure is transmitted from the reservoir to the reader via radio frequency identification technique [16]. The Raumedic Neurovent P-tel probe can telemetrically monitor ICP with the use of 3 primary components: a passive implant, an active antenna, and a corresponding storage and display unit [17]. A piezoresistive pressure transducer is doped at the tip of a polyurethane catheter 33 mm long and 1.67 mm in diameter, which is implanted inside a burr hole with the catheter in the frontal brain parenchyma. Resistors populate a flexible membrane, which directly contacts the pulsating brain tissue. Changes in resistance register within the microchip located in the round ceramic casing; the microchip converts the generated electrical signals into ICP values. This device uses RFID for energy supply and data transmission. RFID activation of the microchip occurs due to the oscillating magnetic field generated by the corresponding TDT1 readP antenna that is connected to the Datalogger MPR-1 monitor [17]. ICP values can be monitored within a frequency range of 1–5 Hz, which is ultimately determined in the settings by the Datalogger user. For ease of data acquisition, data can be transferred and saved to Microsoft Excel.

Published in March of 2020, the Roger’s Research Group through Northwestern University developed a wireless and wearable device for capturing CSF flow rate data in patients with hydrocephalus, which permits the user to back-calculate their ICP [9]. The device has been referred to as a “band-aid sensor,” sitting externally on the skin to measure intermittent and/or continuous flow. The device was constructed using commercial components, utilizing thermal transport data of near-surface skin layers to generate measurements. Using a BLE System and a rechargeable battery, the CSF flow data is transmitted to an Android mobile application that is available to the patient. This device allows readings in a clinical or home setting [9]. Though this device utilizes BLE to transmit pressure data to an application, like our presented device, it does not function without an external wearable component. Additionally, the wearable component limits the feasibility of continuous feedback as environmental factors may limit its utilization. Furthermore, our presented prototype utilizes a piezoresistive pressure sensor integrated within the lining of the catheter walls, which has the potential for increased accuracy with the Millar custom designed sensor, limiting existing errors. 

An additional long-term noninvasive ICP monitoring device has been studied in recent literature which employs photoplethysmography (PPG) technology. The basis of this technology involves near infrared spectroscopy to monitor cerebral oxygenation by shining multiple wavelengths of near-infrared light into the brain. This light reaches the cerebral tissue, which can then be used to indirectly determine changes in cerebral oxygenation. Synchronous pulsations and changes in the morphology of the detected optical signal based on morphology can then be utilized to determine ICP [18]. The in-vitro device utilized in the study by Abay et al. employs an optical custom-made reflectance sensor comprised of a high-intensity light-emitting diode and a silicone photodiode detector. The benefits of this device include noninvasive means of measuring ICP and quick triage of patients presenting with acute conditions that may cause elevated ICP, such as trauma. Furthermore, the PPG device is sensitive to environmental factors such as ambient light, noise, and patient specific physiologic parameters, including body size and position, which may affect its reliability [19,20]. For example, the device relies on the arterial pressure to assess ICP; in certain pathologies such as subarachnoid hemorrhage (SAH) with sequela such as vasospasm, cerebral arterial compression may be a distinct physiological process not accurately reflecting ICPs due to disturbance in autoregulatory mechanisms. Cerebral autoregulation is known to be altered in SAH and similar physiologic states; thus, due to alterations in the relationship between mean arterial pressure (MAP), ICP, and cerebral perfusion pressure (CPP), arterial vasoconstriction detected via the PPG device may not adequately reflect true ICP [21]. 

Our presented device offers a few distinct benefits, such as the ability for patients to measure ICP remotely and provides continuous monitoring on a mobile application without a wearable component, as it is integrated into their existing shunt catheter. Other noninvasive ICP monitors, such as the device by Abay et al., differ as they require a wearable component that may be sensitive to motion artifact contact pressure and require full time application of the wearable component by the patient to obtain continuous data [22]. Additionally, our presented device offers a specific benefit of integration of the pressure sensor into the lining of the proximal catheter, targeting a specific population of shunted hydrocephalus patients and aiding in the workup of shunt failure. Our device aims to provide insight into the specifics of shunt failure, proximal/distal failure, or disconnection, specifics of which may not be attainable with other technologies.

The principal limitations of the pre-existing intracranial pressure monitors include their invasive nature and the inability to transmit pressure data in a wireless manner to a mobile application without an external transducing source, limiting their ability to monitor ventricular shunt function in an ambulatory setting. Other models require a wearable sensor which also limits continuous data collection. Our presented device model is the first of its kind to offer a method of continuous intracranial pressure monitoring utilizing a piezoresistive pressure sensor with transmission to a mobile application without the utilization of an external device. Continuous data collection would allow future applications of this device in the study of relatively poorly understood pathologies such as IIH or normal pressure hydrocephalus by providing real time second to second monitoring of ICP. Additionally, the presented product offers simultaneous data acquisition for the patient as well as the physician with future integrations into the EMR.

A clinical novelty of the proposed device includes its utility to function as an independent non-invasive system interrogation in triaging patients for surgical intervention in the setting of shunt failure. Previously mentioned devices are not integrated into the lining of the proximal catheter. This design feature improves the potential to yield predictable detection of clinical shunt failure. For example, if a patient presented with symptoms of shunt failure and their mobile application continuously demonstrated graphical data similar to Figure 6, Figure 7 and Figure 8, the triaging neurosurgeon could consider a disconnection distal to the pressure sensor, valve failure, or distal catheter disconnection or occlusion; thus, concluding proximal shunt failure to be unlikely. Identification of the malfunctioning hardware utilizing this device may eventually eliminate the invasive aspects of shunt failure workup, including shunt tap and/or exploratory surgery.

Considerations of the current device include margins of error greater than 2 mmHg at pressure ranges greater than 20 mmHg. Future improvements include reduction of this margin of error via integration of microscale customizable Millar pressure sensors, which have the capabilities to prioritize drift and improve error ranges at higher pressure ranges. Future industry changing applications of our presented device include integration into ventriculopleural, ventriculoatrial, lumboperitoneal shunts, and external ventricular drains, all of which employ a catheter system to divert CSF from the ventricular or lumbar subarachnoid space to the pleural space, atrium, peritoneal space or externally, respectively. We are currently designing an integrated pressure sensing technology system with an accessible inner lumen which can then be paired with any brand of shunt valve. 

We would like to note the importance of distinguishing the presented prototype from existing non-invasive ICP monitors. Our device is not marketed as a noninvasive means to measure ICP but rather as a tool to measure ICP in patients with hydrocephalus requiring implanted ventricular shunts to address their underlying pathology, as well as a tool to triage shunt failure and address workup of shunt failure without invasive measures. Many devices, such as tympanic membrane pressure transducers, as well as ocular modalities (optic nerve sheath diameter measurement, ocular sonography, etc.), exist as noninvasive surrogates to estimate ICP [23,24]. These devices have countless clinical applications in patients with trauma, IIH, hydrocephalus, etc., to estimate intracranial pressure without invasive procedures; however, they do not serve as a precise means to work up shunt failure. These devices are not integrated into the components of the shunt system and do not allow for specific triage of a malfunctioning system.

This prototype should serve as a proof-of-concept design for an implantable, continuous, rechargeable ICP-monitoring system. We recognize that the aforementioned considerations limit the current reliability of this prototype and hinder the direct utilization of this study as a validation study for clinical trials. Further ex-vivo studies will be necessary prior to utilizing this device in animal model studies or human clinical trials. These future studies will incorporate an improved iteration of this design with an integrated inner lumen pressure sensor and a greater focus on battery safety by more closely replicating physiologic conditions.

## 5. Conclusions

The presented ex-vivo macro-scale prototype of an implantable, continuous, rechargeable ICP monitoring system has the potential to measure clinically relevant ICPs and assist in the detection of shunt malfunction via wireless communication to a patient’s mobile application. Future work includes microscale prototype development with the integration of a custom piezoresistive pressure sensor with improved drift, and integration of data into patient’s electronic medical records.

## 6. Provisional Patent

Elsawaf, Y. Bluetooth-Compatible Continuous Intracranial Pressure Sensor with Wireless Transmission via Mobile Application. U.S. Provisional Patent, 63/093,154, 16 October 2020.

## Figures and Tables

**Table 1:** Trade study: pressure measurement application, wireless transmission, storage, battery recharging.

**Pressure Measurement**	**Precision (25%)**	**Direct Measurement (10%)**	**Surgical Integration (20%)**	**Durability (5%)**	**Attainability (20%)**	**Power Req (20%)**	**Score**			
Compressed Membrane	5	5	4	5	4	3	4.20			
Light Pulse	3	1	2	4	3	4	2.85			
Thermal Flow	4.5	2.5	4	4.5	4	2	3.60			
Non-thermal Flow	4	2.5	4	4.5	4	2	3.48			
**Wireless Transmission**	**Fully Internal (17.5%)**	**Data Transfer (7.5%)**	**Continuity (17.5%)**	**Speed (7.5%)**	**Transmission Range (12.5%)**	**Security (5%)**	**Power Req (17.5%)**	**Surgical Integration (10%)**	**Accessibility (5%)**	**Score**
Bluetooth	5	4.5	5	4	4	5	3	4.5	5	4.36
Bluetooth Low Energy	5	4	4.5	4.5	5	4	4.5	5	5	4.66
Near Field Communication	1	5	3	4	1	5	5	4.5	5	3.33
Radio Frequency	1	5	5	5	5	2	5	1	3	3.65
**Storage**	**System Integration (40%)**	**Storage Capacity (30%)**	**Security (20%)**	**Cost (10%)**	**Score**					
Cloud	1	5	2	4	2.70					
Internal	4	5	4	5	4.40					
**Battery Recharging**	**Power Delivery (20%)**	**Safety (15%)**	**All Internal (15%)**	**Durability (15%)**	**Size (15%)**	**Charging Distance (10%)**	**Accessibility (10%)**	**Score**		
Subcutaneous Solar Cells	1	3	5	3	3	4	1	2.80		
Qi Charging	5	5	1	5	3	4.5	3	3.85		
Radio Frequency	2	1	1	5	5	5	2	2.90		
Thermoelectric	1	5	5	3	4	5	2	3.45		
Piezoelectric	1.5	3	5	3	4.5	5	1	3.23		

**Table 2:** Accuracy testing data.

Pressure (mmHg)	0	5	10	15	20	25	30	40	60	80	100
Rep 1 (SD)	0.09 (0.07)	4.03 (0.13)	10.77 (0.86)	17.11 (0.28)	22.66 (0.15)	28.99 (0.14)	34.83 (0.20)	44.45 (0.55)	68.12 (0.53)	94.12 (0.99)	1117.33 (0.20)
Rep 2 (SD)	−0.44 (0.11)	7.73 (0.59)	8.95 (0.22)	12.13 (0.12)	17.34 (0.41)	22.23 (0.20)	28.54 (0.38)	38.33 (0.19)	59.37 (0.17)	84.55 (0.33)	108.86 (0.29)
Rep 3 (SD)	−0.44 (0.04)	5.6 (0.13)	9.22 (0.22)	15.43 (0.12)	21.04 (0.10)	27.38 (0.20)	32.58 (0.17)	44.13 (0.19)	66.47 (0.18)	91.39 (0.11)	115.86 (0.16)
Rep 4 (SD)	0.38 (0.25)	5.45 (0.15)	10.22 (0.28)	16.04 (0.12)	20.95 (0.20)	26.76 (0.12)	32.28 (0.23)	44.31 (0.16)	66.6 (0.18)	90.18 (0.33)	113.11 (0.15)
Rep 5 (SD)	−0.72 (0.12)	5.09 (0.26)	13.2 (1.48)	17.55 (0.14)	21.78 (0.12)	29.11 (0.24)	33.5 (0.17)	44.68 (0.22)	68.34 (0.20)	91.52 (0.52)	114.92 (0.48)

## Data Availability

Data are contained within the article and Appendix A and Appendix B.

## Appendix A. Supplementary Methods

### Trade Study and Design Solutions

A representative trade study was initially conducted to evaluate the most optimal design components for pressure sensing methods and location, methods of wireless transmission, powering options, storage capabilities, and integration of the system components, as well as other logistical elements such as durability and attainability. Various methods of pressure sensing, such as compressed membrane, light pulse, and thermal and non-thermal flow, were considered. Compressed membrane sensors measure the deformation of a diaphragm or membrane acted on by surrounding forces and convert that distortion into an electrical signal [25]. Light pulse sensors send electromagnetic waves through a catheter to a detector to measure flow [26]. In contrast, thermal flow sensors indirectly measure the velocity of fluid near the wall of a tube via the alteration of a temperature profile to computationally determine flow [27]. Non-thermal flow sensors, like the acoustic-ultrasonic flow meter shown, use the differential transit times of ultrasonic signals as affected by the fluid in the tube to determine the flow. Of note, all included sensors outputting flow measurements can accurately back-calculate pressure gradients in a system [28]. The trade study determined a compressed membrane to be the most ideal (Table 1); the precision of compressed membrane components investigated read within 0.1% error, far below the design requirement threshold. Measurement of compressed membrane sensors does not require calculations, as voltage output corresponds linearly with measured pressure in the surrounding environment, thus receiving a 5/5 for direct measurement [29]. Surgical integration was rated a 4/5, as the device can integrate into the wall of the proximal catheter of the VP shunt. Durability was rated a 5/5 based on two standards: components were found to withstand extreme forces far surpassing the subcutaneous physiological environment and were awarded an anticipated lifetime on the scale of decades [30]. Attainability was rated a 4/5 due to ease of purchase. Power requirements were rated as a 3/5, as a voltage on the scale of 2 to 4 Volts was necessary to power the device. These values averaged to a total of 4.20/5 based on the weightings of each of the design considerations. A 2FMV microelectromechanical (MEMS) Connect 100 Vented Millar, Inc. Piezoresistive Sensor with a 0.67 mm outer diameter was selected for the pressure sensing component of the design.

A second trade study was conducted to determine methods of wireless transmission; Bluetooth low energy (BLE) was selected as the most ideal (Table 1). BLE consists of a Controller, which is usually a small System-on-Chip containing a radio, and a Host, which runs on an application processor chip; the Host Controller Interface manages communication between the Host and Controller [11]. In the proposed device, the Controller would be implemented in our pressure sensor, and the Host would be the iPhone [31]. Bluetooth low energy was ranked 5/5 for being fully internal, as it does not require an external component to be used. It was ranked 4.5/5 for data transfer capacity because it can transmit a maximum of 47 bytes, which is less than Bluetooth, which transfers 1 Mb of data per second [32,33]. However, both forms of Bluetooth connectivity are sufficient for the predetermined design requirements. BLE was ranked 4/5 for security for the 128 bit Advanced Encryption Standard, but it does not require authentication for Apple devices. BLE was ranked 5/5 for transmission range because it can transmit data for over 50 m [32]. For power requirements, BLE was awarded 4.5/5 because it requires less than 15 mA [32] on average, which is 0.01–0.5× less energy than Bluetooth of energy [34]. BLE can be powered using the energy of a small battery or a “button battery” for 4–5 years [32,35]; thus, the proposed device could theoretically withstand the lifetime of the shunt without requiring charging. BLE remains in “sleep mode” at baseline and only utilizes power when a connection is initiated [34]; because of this intermittent connection, BLE was ranked 4.5/5 for continuity. However, BLE still scored highly for continuity because the intermittent connection can transmit multiple times per second, therefore meeting the design requirements. BLE was ranked 5/5 for surgical integration because it is commonly used for monitoring devices and wearable devices [35], such as blood pressure monitoring [33]. Additionally, BLE devices range from 2.5 × 2.5 × 1 mm–6 × 6 × 1 mm [36], which met size requirement to integrate within the system. Finally, BLE was ranked 5/5 for accessibility, as it can be easily purchased online. These values averaged to a total of 4.66/5 based on the weightings of each of the design considerations.

A trade study was then conducted to determine storage capabilities (Table 1); internal storage was determined to be most ideal. Internal storage stores pressure sensor data as a file in a memory chip that can be periodically locally accessed via Bluetooth Low Energy [37]. Specifically, electrically erasable programmable read only memory (EEPROM) is most applicable since it can read and store frequently updated files [38]. For system integration, internal storage was given a 4/5 since there is a common code for converting sensor data into storage on the SD card and for accessing the data via BLE [36]. Internal storage chips are available in microSD sizes (11 mm × 15 mm × 1 mm) for easy incorporation into the housing of the device for the pressure sensor and other electrical components [39]. Additionally, flash memory drives are not constructed from magnetic materials and, therefore, would not be altered by MRI induced magnetic fields [40]. For data storage capacity, internal storage was rated a 5/5 as microSD cards are available with up to 256 GB of storage capacity, far exceeding the design requirement of approximately 54 MB of data [41]. Internal storage was rated a 4/5 for security due to the unlikeliness of security breaches of an SD card once it has been implanted. Lastly, when considering cost, internal storage was rated a 5/5 due to the ease of attainability. Overall, based on the weights of our design considerations, internal storage was rated a 4.4/5.

A final trade study was conducted to determine powering options (Table 1) for which Qi charging was determined most ideal. Qi is an established method of wireless power transmission which utilizes inductive coupling [11]. In 2014, S. Hached and colleagues proposed a BLE-compatible Qi battery for use in implantable medical devices that is composed of a Qi receiver, a BLE module, and a charging circuit. To charge an implanted device using this proposed battery, a patient aligns the external Qi transmitter coils in the wireless charger to the implanted Qi receiver coils in the device. This alignment allows the two coils to establish a connection and transfer power into the subcutaneous Qi receiver, which could then be used to power the rest of the implanted device [11]. This charging modality has since been widely implemented in many health monitoring devices, such as wearable heart rate monitors [12]. Qi charging was ranked 5/5 for power delivery, as it has been established to provide on the scale of 15 W for standard devices and, in extreme cases with newer models, up to 30–60 W [42]. It was ranked a 5/5 for safety, as extensive evidence supports Qi charging utilization for medical device implementation in the past, with minimal potential bodily harm [13]. It was ranked a 1/5 on the all-internal category, as a secondary external component is required to recharge the internal battery. It was ranked 5/5 for durability, as evidenced by the extensive medical device applications [13]. Additionally, the external Qi charging component can be replaced without extensive surgical intervention. Qi charging was ranked a 3/5 for size, as rectangular coils on the external component can be on the order of 30–40 mm in typical models; however, this would not have to be incorporated internally, and the coil size could potentially be modified given the system’s low power needs. Qi charging distance was given a 4/5, as the standard confidence range is approximately 4 cm, which would allow for subcutaneous charging [14]. Finally, accessibility was ranked a 3/5, as Qi wireless chargers are readily available but may require modifications to reduce coil density to accommodate size [13]. These values averaged to a total of 3.85/5 based on the weightings of each of the design considerations.

## Appendix B. Supplementary Data

Average attained pressure values for each theoretical pressure in Protoype Modeled ICP Detection from Section 3.

Average attained pressure for a theoretical pressure of 0 mmHg was −0.226 mmHg ± 0.351. Average attained pressure for a theoretical pressure of 5 mmHg was 5.58 mmHg ± 1.058. Average attained pressure for a theoretical pressure of 10 mmHg was 10.472 mmHg ± 1.328. Average attained pressure for a theoretical pressure of 15 mmHg was 15.652 mmHg ± 1.678. Average attained pressure for a theoretical pressure of 20 mmHg was with a standard deviation of 20.754 ± 1.59. Average attained pressure for a theoretical pressure of 25 mmHg was 26.894 mmHg ± 2.193. Average attained pressure for a theoretical pressure of 30 mmHg was 32.346 mmHg ± 1.841. Average attained pressure for a theoretical pressure of 40 mmHg was 43.18 mmHg ± 2.131. Average attained pressure for a theoretical pressure of 60 mmHg was 65.78 mmHg ± 2.888. Average attained pressure for a theoretical pressure of 80 mmHg was 90.352 mmHg ± 2.781. Average attained pressure for a theoretical pressure of 100 mmHg was 114.016 mmHg ± 2.559.
